# A Compact Low-Power Chopper Low Noise Amplifier for High Density Neural Front-Ends

**DOI:** 10.3390/s25041157

**Published:** 2025-02-13

**Authors:** Alessandro Fava, Francesco Centurelli, Pietro Monsurrò, Giuseppe Scotti

**Affiliations:** Department of Information, Electronics and Telecommunication Engineering, Sapienza University of Rome, 00184 Roma, Italy; alessandro.fava@uniroma1.it (A.F.); francesco.centurelli@uniroma1.it (F.C.); pietro.monsurro@uniroma1.it (P.M.)

**Keywords:** low noise amplifier, chopping, neural recording, bio-amplifier, analog front-end

## Abstract

This paper presents a low-power and area-efficient chopper-stabilized low noise amplifier (CS-LNA) for in-pixel neural recording systems. The proposed CS-LNA can be used in a multi-channel architecture, in which the chopper mixers of the LNA are exploited to provide the time division multiplexing (TDM) of several channels, while reducing the flicker noise and rejecting the Electrode DC Offset (EDO). A detailed noise analysis including the effect of the chopper stabilization on flicker noise, and a design flow to optimize the trade-off between input-referred noise and silicon area are presented, and utilized to design the LNA. The adopted approach to reject the EDO allows to tolerate an input offset of ±50 mV, without appreciably affecting the CS-LNA performance, and does not require an additional DC Servo Loop (DSL). The proposed CS-LNA has been fabricated in a 0.13 μm CMOS process with an area of 0.0268 mm^2^, consuming about 2 μA from a 0.8 V supply voltage. It achieves an integral noise of 4.19 μVrms (2.58 μVrms) from 1 to 7.5 kHz (from 300 to 7.5 kHz) and results in a noise efficiency factor (NEF) of 2.63 (1.62). Besides achieving a maximum gain of 38.67 dB with a tuning range of about 12 dB, the neural amplifier exhibits a CMRR of 67 dB. A comparison with the recent literature dealing with in-pixel amplifiers shows state-of-the-art performance.

## 1. Introduction

One of the main challenges in recent neuroscience research is the monitoring of neural activity with very high temporal and spatial resolutions [1,2,3]. The golden standard for neurophysiology applications is nowadays represented by high-density CMOS neural probes [4,5], which allow for unprecedented recordings of large numbers of neurons at single-cell resolution in animals such as rodents [6], non-human primates [7], and humans [8]. The continuous scaling in terms of number of processed neurons of these architectures requires readout circuits that are more and more efficient, both in terms of noise performance and power density, by allowing, on the one hand, to detect the small signals inside the neuron and, on the other, to avoid brain damage due to device self-heating [9,10] and probe insertion during neurological surgery [11]. In fact, in chronic implantation, the device inserted induces tissue inflammation, causing a cellular sheath formation by glial scar encapsulation, thus isolating neurons from microelectrodes [12]. Considering these surgical issues, the shape, dimensions, surface of the electrodes, and materials used are crucial features in the design of chronic devices. For instance, the widely used electrode coating based on Ag-AgCl leads to biofouling, making it cytotoxic and, in the long term, results in depolarization loss, causing voltage drift and Electrode DC Offset (EDO) over time. Other biocompatible materials, such as Pt-Ir, are preferred, due to their polarization features and lower EDO. In fact, the adoption of Pt-Ir coating allows to achieve measured values of the EDO in the range of 0.1 mV, with a standard deviation of only 2 mV, without significant voltage drift [13].

Neural recording systems using multiplexing techniques to enhance the pixel density have been recently proposed. A prototype of multi-electrode array (MEA) neural recording architecture for Micro-Electrocorticography (μECoG) applications has been presented in [14]. The analog front-end (AFE) integrates 24,320 recording sites, and the prototype presents 380 column-parallel readout channels managing 32 pixels with the time division multiplexing (TDM) approach. The proposed MEA architecture has an active area of 7.65 mm^2^ and allows for a very high-resolution recording. A polyimide-based neural probe has been proposed in [15]. The prototype of 256 recording sites is designed with four-shank flexible probes with a non-standard CMOS process. Each polyimide shank integrates 64 passive microelectrodes and the CMOS front-end is linked to each shank with a hybrid interconnection process and posed away from the recording site. Each shank covers an active area of 1.03 mm^2^.

High-density CMOS neural probes have begun to be commercialized in preclinical and clinical applications. The single-shank of Neuropixel probes developed by IMEC [4], and the four-shank based on SiNAPS technology developed by IIT [6] are two examples of commercially available high-density probes. The four-shank solution by IIT contains an array of 1024 electrode-pixels that are time division multiplexed to only 32 output pads. In these probes, an amplifier is integrated underneath each single electrode enabling the continuous recording at sub-millisecond resolution from all the integrated electrodes. The Neuropixel 1.0 probes contain 384 channels in 45 mm^2^, the four-shank Neuropixel 2.0 probes integrate 384 channels in 19 mm^2^, and it is expected that next generation probes will contain more than 1000 channels in less than 10 mm^2^ [9]; therefore, the area allowed for in-pixel amplifiers, which is nowadays in the range from about 0.05 mm^2^ to about 0.11 mm^2^, is expected to be reduced below 0.01 mm^2^ in the very next future. In the new implantable devices, to cope with all the electrical constraints imposed by large-scale integration, all of the multichannel systems proposed in the literature require noise levels in the range of a few microvolts to detect the action potentials (AP) in the bandwidth from 300 Hz to 7.5 kHz, and the local field potential (LFP) in the bandwidth from 0.5 Hz to 300 Hz, respectively, ensuring in all cases that the EDO is rejected. For all the above reasons, neuro electronics research has undergone a technological evolution in the last decade, proposing different Analog Front-Ends (AFEs) [16,17,18,19,20,21], in which performance has grown in terms of number of recording sites by optimizing the power consumption and noise efficiency of the architectures. Most neural recording architectures employ AC-coupled AFEs [22,23,24], where the recording site area depends exclusively on the size of the input bypass capacitors and the input transistors of the LNA, which have to be sufficiently large to reduce the thermal and flicker noise in the AP and LFP bands.

Switching techniques, such as autozeroing [25] and chopping [26,27,28], are frequently exploited in neural recording front-ends to mitigate flicker noise and to optimize the trade-off between area occupancy and noise floor level.

Conventional chopper stabilized (CS) amplifiers for biopotential acquisition need a dc-servo loop (DSL) to filter the EDO, and even though the addition of the DSL can have a negative impact on noise performance at the relevant frequencies, this degradation is frequently disregarded. An extensive noise study of CS amplifiers with analog DSLs is presented in [29].

In this paper, we present a chopper-stabilized low noise amplifier (CS-LNA) for in-pixel neural recording systems, in which the chopper mixers of the LNA allow to provide the time division multiplexing (TDM) of several channels, while reducing the flicker noise and rejecting the EDO. The capability of rejecting the EDO without a DSL results in a noise improvement of the proposed CS-LNA with respect to conventional chopper amplifiers with DSL. The proposed CS-LNA achieves state-of-the-art performance with a very good trade-off in terms of noise efficiency, area, and power consumption.

In the following, Section 2 presents the architecture of the neural AFE, introducing the principles of the chopper-based TDM, and of the adopted EDO rejection approach. Section 3 deals with the proposed CS-LNA topology and includes a detailed noise analysis highlighting the trade-off between area and noise allowed by the CS-LNA. Section 4 presents the design of the CS-LNA prototype in a 130 nm CMOS technology, Section 5 reports the experimental results, and Section 6 concludes the paper.

## 2. High-Density Neural Front-End Architecture and EDO Rejection Approach

**Figure 1:**
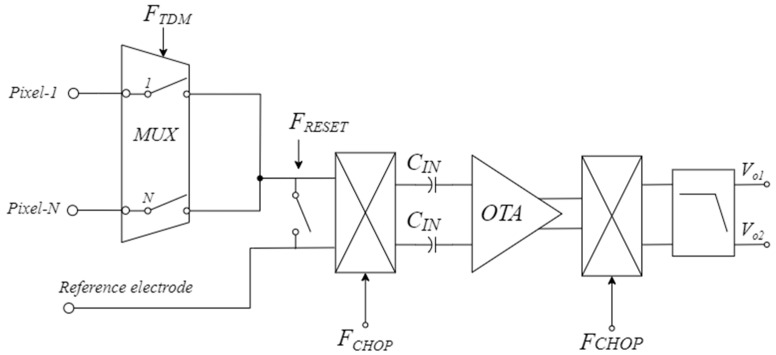
Block scheme of the proposed high-density neural front-end exploiting chopper-based time division multiplexing (TDM).

The proposed CS-LNA has been developed for a high-density neural front-end architecture as reported in Figure 1.

It exploits combining two switching techniques to provide the time division multiplexing (TDM) of several channels while reducing the flicker noise and rejecting the EDO. More specifically, the adopted TDM approach allows for the development of neural channels processing a certain number (N) of pixels, while at the same time providing a servo-loop-free way to cancel the EDO in a similar way as in [30,31,32]. The process that involves the DC offset rejection is explained by the charge sharing between the input equivalent capacitance of the CS-LNA and the capacitance of electrode-electrolyte interface during toggling of the chopper.

Since, as discussed in [30], the time division multiplexing of N channels reduces by N the time duration of the charge sharing process, a reset switch driven by the signal *F_RESET_* in Figure 1 has been inserted between the sensing input of the CS-LNA and the reference electrode to improve the effectiveness of the EDO canceling process.

**Figure 2:**
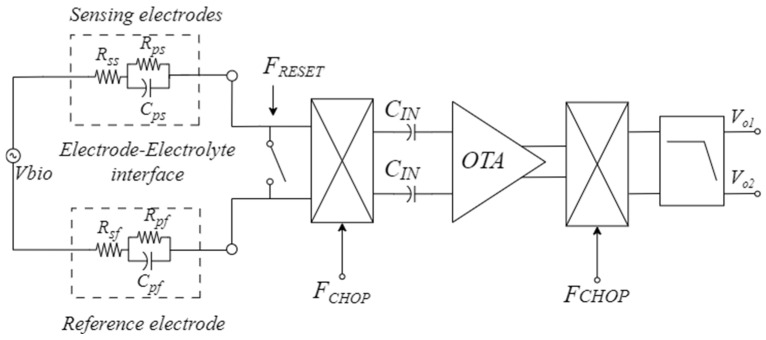
Block scheme with the electrode-electrolyte interface model.

To better explain the working principle of the adopted EDO rejection technique, we have reported in Figure 2 a detail of the interface between the electrodes and the input chopper mixer of the CS-LNA (considering the reference electrode and the *Pixel i* selected by *F_TDM_*), in which the electrode–tissue interface model presented in [30,31] is considered. The resistance of the solution is represented by *R_S_* while the interface capacitive impedance and the charge transfer resistance are modeled with *C_P_* and *R_P_*, respectively. Parameters of the sensing electrode are denoted as *R_SS_*, *R_PS_*, and *C_PS_*, and parameters of the reference electrode are denoted as *R_PF_*, *R_SF_,* and *C_PF_*.

During the reset phase, the switch driven by *F_RESET_* in Figure 2 is closed, and the differential charge stored on the input capacitances of the CS-LNA is moved to the electrode-electrolyte capacitances and dissipated through Rp with a time constant $\tau =R_{P}C_{P}$. In this way, the input DC voltage at the sensing electrode interface converges to the voltage at the interface of the reference electrode, canceling the differential offset.

## 3. Proposed CS-LNA

In this section we present the proposed CS-LNA by introducing the topology, the noise analysis and optimization and a comprehensive design flow.

### 3.1. Preliminary Considerations

To date, many neural amplifier architectures proposed in the literature are based on the well-known Capacitive Feedback Network (CFN) topology [16]. It is an AC-coupled closed-loop architecture in which the gain is established by the ratio of the input and feedback capacitances. Closed-loop amplifiers are often used in analog applications because they provide a voltage gain, which can be accurately set by the ratio of two passive elements, and results very stable under process, supply voltage, and temperature (PVT) variations. Another important feature of closed-loop amplifiers is that their linearity performance is improved by the feedback mechanism. Since, in CFN, amplifiers the gain is set by the ratio of two capacitors, they require a high area footprint to implement large gains. It has to be noted also that closed-loop amplifiers often need more than one stage to boost the direct-path gain and achieve a closed-loop gain in the range of 35–40 dB. This leads to the need of Miller compensation for stable operation, which results in additional area occupation, and to a higher number of current branches, which results in high power consumption. Furthermore, since the input-referred noise (IRN) of the front-end is filtered by the inverse of the transfer function of the amplifier, as well analyzed by Chaturvedi et al. in [24], closed-loop amplifiers exhibit higher IRN than open-loop amplifiers.

Recently, the neurosciences community has proven that information is strongly encoded in the time-spatial distribution of the spikes [27] and statistical analyses, such as firing rate [33,34,35], have been carried out to gain a deeper insight of the processes inside the brain. The results of these activities have shown that gain errors due to distortions or PVT variations in electrical parameters do not have a great impact on the information provided by the acquired neural signals. Furthermore, in new-generation probes, in which a high spatial resolution allows us to record the AP in a single neuron, the accuracy of the gain is not the essential specification to permit realizing a compact pixel, and ultra-low-noise performance, small area, and minimal power consumption are the most important requirements [24].

A single-stage open-loop amplifier can be used to achieve the needed voltage gain without the need of feedback capacitors, thus avoiding frequency compensation, and minimizing the number of current branches, thus requiring less silicon area and less power consumption than a conventional closed-loop amplifier. Even if open-loop amplifiers exhibit larger distortions than closed-loop amplifiers, this, as pointed out in [27,35], is not so important for an in-pixel neural recording LNA, as the input voltage swing is limited. Another important limitation of open-loop amplifiers is that their main performance parameters, such as gain and bandwidth, result more sensitive to PVT variations than in closed-loop configurations. However, the effects of PVT variations can be compensated by implementing programmable gain amplifiers and exploiting digital calibration approaches in the postprocessing of the neural spikes.

### 3.2. Topology of the Proposed CS-LNA

In conventional AC-coupled LNAs, the acquisition efficiency of the signals depends on the minimization of input-referred noise (IRN), which demands a large power consumption to maximize the transconductances of transistors in the input differential stage. The chopper stabilization (CS) technique [26] is often used to reduce the flicker noise by modulating the neural signal at a higher frequency. In the proposed CS-LNA, we combine the CS approach with an open-loop AC-coupled LNA in order to allow an optimal trade-off between the IRN and the transconductance of the input differential pair, thus reducing area and power consumption with respect to previously reported neural amplifiers.

The open-loop approach provides a compact low-power amplifier, at the cost of some sensitivity to PVT variations. On the other hand, the use of chopping makes the effect of flicker noise negligible, thus allowing to optimize the design by taking into account just the white noise contribution. The adoption of the DSL-free EDO rejection approach presented in Section 2 further improves the noise performance. With a careful sizing and biasing, all these features allow designing a single-stage low-noise amplifier suitable for high-density AFE applications.

**Figure 3:**
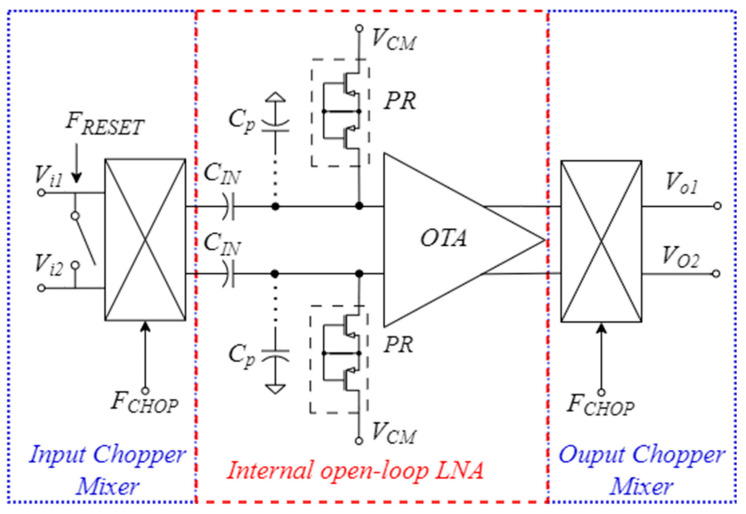
The proposed LNA architecture with input and output chopper mixers.

**Figure 4:**
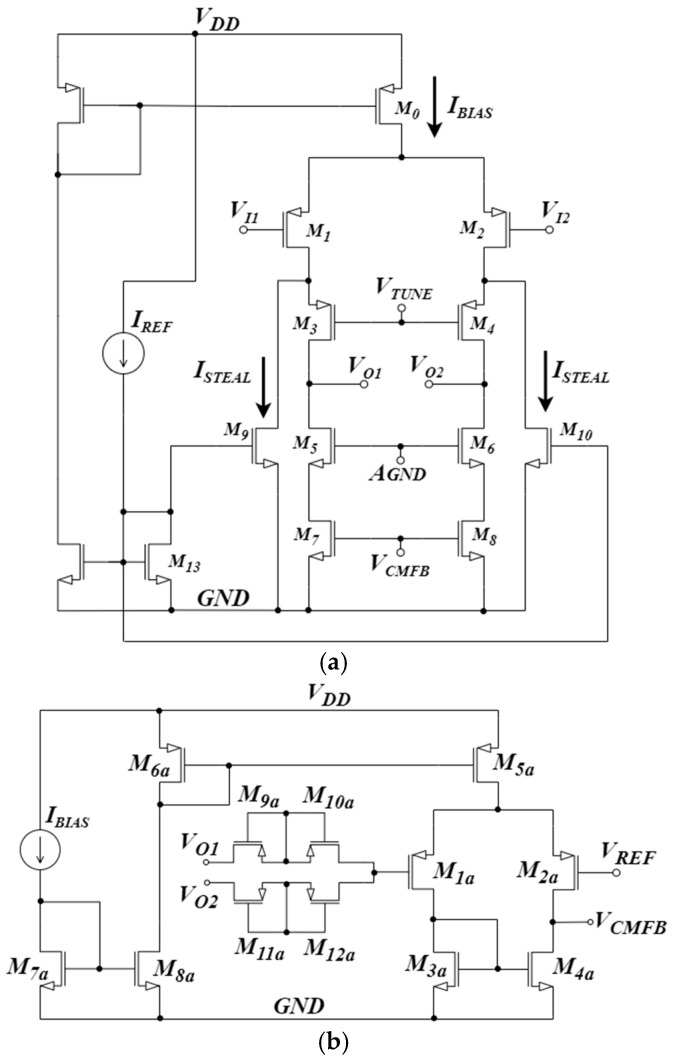
OTA architecture: (**a**) schematic of OTA that uses auxiliary MOSFETs to steal current at output branch to maximize the output resistance and increase the voltage gain; (**b**) the common-mode feedback (CMFB) loop that exploits two pseudo-resistors as common mode estimator.

**Figure 5:**
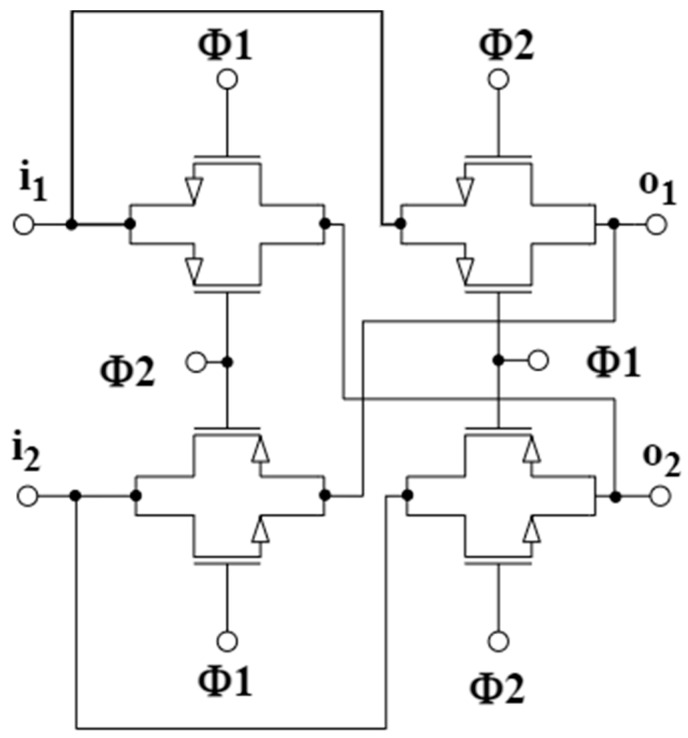
Transistor-level implementation of chopper mixers.

The block scheme of the proposed CS-LNA is presented in Figure 3, where *C_IN_* denotes the input bypass capacitors which, together with the equivalent resistance of the pseudo-resistors *PR* set the low cutoff frequency f_L_ of the amplifier, and *C_p_* are parasitic capacitances (including both the input capacitance of the OTA and the layout parasitic capacitances). The operational transconductance amplifier (OTA) block in Figure 3 has been implemented through the open-loop topology reported in Figure 4, whereas the chopper mixers have been implemented by using four crossed transmission-gate (TG) switches, clocked with opposite phases, as shown in Figure 5. The topology of the OTA in Figure 4 is similar to the OTA presented in [24], but in the proposed circuit the PMOS load has been replaced by an NMOS cascode current source implemented by (M_5_–M_8_) to provide higher gain and allow an improved common-mode rejection thanks to the CMFB loop reported in Figure 4b, which is closed at the gate terminals of M_7_ and M_8_. Current sources M_9_ and M_10_ allow us to subtract the current $I_{STEAL}$ from the current flowing in M_1_ and M_2_, thus increasing the output resistance of the cascode current source and, thus, the maximum achievable voltage gain.

### 3.3. Noise Analysis and Optimization

#### 3.3.1. Noise Analysis and Optimization of the Internal LNA

In this section, we study the noise of internal open-loop LNA reported in Figure 3, whereas in Section 3.2, we will focus on the effect of the chopper mixers (neglecting the noise contribution of the switches).

To analyze the noise of the internal open-loop LNA, we refer to Figure 3, which highlights the parasitic capacitances *C_p_* at the input of the OTA, and neglect the effect of the chopper mixers. The noise power $V_{n,in,LNA}^{2}$, referred to the output of the input chopper mixer, can be computed [24] starting from the input-referred noise power spectral density (PSD) of the OTA $S_{n,in,OTA}$, by considering the voltage divider effect due to the capacitances *C_IN_* and *C_p_*, and integrating over the working bandwidth ($F_{H}-F_{L}$),

(1) $$V_{n,in,LNA}^{2}=\int_{F_{L}}^{F_{H}}\frac{S_{n,in,OTA\left(f\right)}}{{\left|H_{Cdiv}\left(f\right)\right|}^{2}}\;df={\left(\frac{C_{IN}+C_{p}}{C_{IN}}\right)}^{2}\int_{F_{L}}^{F_{H}}S_{n,in,OTA}\left(f\right)\;df$$

The low cutoff frequency $F_{L}$ is in the order of 1 Hz in typical neural recording applications.

The input-referred PSD of the OTA includes both thermal and flicker noise contributions and, with reference to Figure 4, it can be written as

(2) $$S_{n,in,OTA}\left(f\right)=S_{n,in,th}\left(f\right)+S_{n,in,fl}\left(f\right)$$

(3) $$S_{n,in,th}\left(f\right)=8k_{b}T\gamma \left(\frac{1}{gm_{1,2}}+\frac{gm_{7,8}}{gm_{1,2}^{2}}+\frac{gm_{9,10}}{gm_{1,2}^{2}}\right)$$

(4) $$S_{n,in,fl}\left(f\right)=\frac{2K_{f}}{C_{ox}W_{1,2}L_{1,2}}\frac{1}{f}+\frac{2K_{f}}{C_{ox}W_{7,8}L_{7,8}}\frac{1}{f}\frac{gm_{7,8}}{gm_{1}^{2}}+\frac{2K_{f}}{C_{ox}W_{9,10}L_{9,10}}\frac{1}{f}\frac{gm_{9,10}}{gm_{1,2}^{2}}$$

where a factor 2 is due to the fully differential architecture, γ is the thermal noise coefficient and depends on the effective mobility of transistors, $k_{b}$ is Boltzmann constant, and *T* is the temperature in Kelvin. $K_{f}$ is a technology parameter of flicker noise, and $C_{ox}$ is the oxide capacitance per unit gate area.

Equations (2)–(4) show that the noise contribution of transistors other than that of the input differential pair M_1_–M_2_ can be minimized by designing the OTA with

(5) $$gm_{i}\ll gm_{1,2}\;\;i\;=\;3\ldots 10$$

This is usually achieved by decreasing the transistors aspect ratio for a given bias current, with respect to the input pair devices. However, this approach is limited by technological constraints on minimum and maximum sizes of MOS devices in a specific CMOS process, and by the allowable supply range.

More specifically, since the current sources M_9_ and M_10_ subtract the current $I_{STEAL}$ from the current flowing in M_1_ and M_2_, the contributions of transistors from M_3_ to M_8_ is negligible. Furthermore, even if the bias current flowing in M_9_ and M_10_ is similar to the bias current in M_1_ and M_2_, their dimensions can be designed to lower $gm_{9,10}$ with respect to $gm_{1,2}$, thus reducing their noise contribution. Therefore, if a careful sizing of MOS transistors is carried out, the input-referred noise of the OTA is dominated by the input pair devices. PMOS transistors are adopted for the input pair, due to their lower noise contribution [33]; their size and aspect ratio have to be chosen taking into account both noise and the other requirements such as gain, power consumption and the minimization of silicon area.

The latter involves an essential trade-off in the design of the LNA. Area is typically dominated by the capacitance *C_IN_*, that is large to set a low cutoff frequency. If such capacitor is reduced to fulfill area requirements, the effect of the parasitic capacitance *C_p_* results in a reduction in the mid-band gain (due to the divider effect) and consequently in an increase in the input-referred noise, as shown by Equation (1).

The parasitic capacitance *C_p_* is mostly due to the input capacitance of the OTA, thus it is proportional to the gate area of the input pair devices $W_{1,2}\;L_{1,2}$. Reducing its effect therefore implies reducing the size of M_1_-M_2_; on the other hand, this increases the flicker noise contribution (4), that is the dominant contribution to (2).

To solve this trade-off, the proposed LNA exploits the chopper technique [36,37], which allows for removing the flicker noise, so that the size of M_1_–M_2_ can be reduced to minimize the parasitic capacitance *C_p_*, and only thermal noise has to be optimized.

#### 3.3.2. Noise Analysis and Optimization of the Chopper Stabilized LNA

The chopper stabilization (CS) technique [38,39] is a well-known approach used to push flicker noise out the band of the amplifier, thus improving the signal-to-noise ratio for low frequency signals.

**Figure 6:**
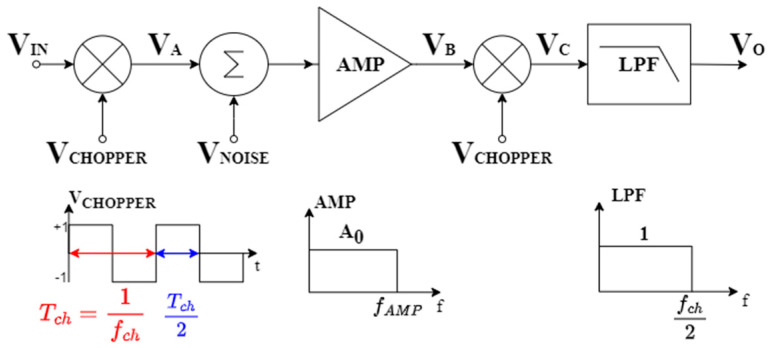
The signal processing of the chopper stabilization technique.

In this section, the noise analysis of a chopper-stabilized amplifier is revised to set the ground for the optimized design flow we discuss in the next subsection. For this purpose, it is necessary to calculate the noise at the output of the chopper architecture by accounting for the signal processing at the different intermediate stages, as reported in Figure 6.

To simplify the analysis, we assume ideal chopper mixers and ideal (brick wall) low-pass (LP) filter behavior both for the amplifier and the low-pass filter (LPF), as in [38,39,40].

The gains and bandwidths of the ideal amplifier and LP filter are denoted as $A_{0}$, $f_{AMP}$ and 1, $f_{ch}/2$ respectively. The symmetrical-square waveform sources *V_CHOPPER_* with frequency $f_{ch}$ drive the chopper mixers at the beginning of the processing chain and after the amplifier stage. The first mixer aims to translate the signal around the frequency $f_{ch}$, while the second mixer is responsible for bringing the signal back to baseband and modulating the noise introduced by the amplifier at the frequency $f_{ch}$. The signal *V_CHOPPER_* can be expressed in the frequency domain as follows:

(6) $$V_{CHOPPER}\left(f\right)=\frac{2}{\pi }\sum_{k=-\infty \;odd\;}^{+\infty }\frac{1}{jk}\delta (f-kf_{ch})$$

To conduct the noise analysis, the signal spectrum at the output of each stage has to be computed, and the spectra of the signals at nodes *V_A_*, *V_B_*, and *V_C_* can be written as follows:

(7a) $$V_{A}\left(f\right)=\frac{2}{\pi }\sum_{k=-\infty odd}^{+\infty }\frac{1}{jk}V_{IN}(f-kf_{ch})$$

(7b) $$V_{B}\left(f\right)=\frac{2}{\pi }\sum_{k=-N\;odd}^{+N}\frac{A_{0}}{jk}V_{IN}\left(f-kf_{ch}\right)+N_{AMP}\left(f\right)\;A_{0}rect_{f_{AMP}}(f)$$

(7c) $$V_{C}\left(f\right)=-\frac{4}{\pi^{2}}\sum_{n=-N\;odd}^{+N}\sum_{k=-N\;odd}^{+N}\frac{A_{0}}{n*k}V_{IN}\left(f-kf_{ch}-nf_{ch}\right)+\frac{2}{\pi }\sum_{n=-N\;odd}^{+N}\frac{1}{jn}A_{0}N_{AMP}\left(f-nf_{ch}\right)$$

In Equation (7c), the limits of the summation, denoted as *N*, are defined as the number of spectral replicas of the modulation within the bandwidth of the amplifier, precisely $N=f_{AMP}/f_{ch}$, whereas $N_{AMP}$ represents the RMS noise signal (computed as the square root of the PSD) introduced by the amplifier and modulated by the second mixer.

The spectrum at the output of the LP filter is reported in Equation (8) and has been decomposed into its signal and noise components, respectively. For the signal component Equation (8b), given that the filter is defined up to half the Nyquist frequency, the only spectral component present is that of the baseband signal obtained for indices n=−k from Equation (7c). In Equation (8b), $\zeta_{F}$ is defined as the finite series of the Riemann zeta function at 2 (ζ(2)) in which only odd indices are considered. As for the noise component in Equation (8c), both thermal and flicker noise have been taken into account in $N_{AMP}$.

(8a) $$V_{O}\left(f\right)={V_{O}}_{sig}\left(f\right)+{V_{O}}_{noise}\left(f\right)=V_{c}\left(f\right)\cdot H_{LPF}\left(f\right)$$

(8b) $$V_{O_{sig}\;}\left(f\right)=-\frac{4}{\pi^{2}}A_{0}\sum_{n=-N\;odd}^{+N}\frac{1}{n^{2}}\;V_{IN\;}\left(f\right)=-\frac{8}{\;\pi^{2}}A_{0}\;\zeta_{F}{\;V}_{IN}(f)$$

(8c) $$V_{O_{noise}}(f)=\frac{2}{\pi }\sum_{n=-N\;odd}^{+N}\frac{1}{jn}A_{0}N_{AMP}\left(f-nf_{ch}\right)$$

(9) $$\sum_{n=-N\;odd}^{+N}\frac{1}{n^{2}}=2\;*\;\sum_{n=1\;odd}^{+N}\frac{1}{n^{2}}=2\cdot \zeta_{F}$$

To assess how much noise is folded into the baseband, it is necessary to express the PSD of the amplifier noise as follows:

(10) $$S_{AMP}\left(f\right){=S}_{th}\left(f\right)+S_{flicker}\left(f\right)=N_{th}(1+\frac{f_{nc}}{\;|f|})\;$$

where *N_th_* is the white thermal noise spectral density and $f_{nc}$ is the noise corner frequency of the amplifier. Starting from (10), the PSD of the output noise of the overall LNA can be expressed as

(11) $$S_{N_{0}}(f)=\frac{4}{\pi^{2}}A_{0}^{2}\sum_{n=-N\;odd}^{+N}\frac{1}{n^{2}}S_{AMP}\left(f-nf_{ch}\right)$$

Since, in the application of interest, we can assume that $f_{nc}<f_{ch}/2$, it is possible to neglect the flicker noise in the folding of noise into the first Nyquist band and determine the PSD at the output of the chopper architecture as follows:

(12) $$S_{N_{0}}\left(f\right)=\frac{4}{\pi^{2}}A_{0}^{2}\sum_{n=-N\;odd}^{+N}\frac{1}{n^{2}}\;N_{th}\;=\frac{8}{\pi^{2}}A_{0\;}^{2}N_{th\;}\zeta_{F}$$

Equation (12) allows highlighting the advantages of the chopper approach. By comparing Figure 3 and Figure 6, it is clear that $S_{AMP}\left(f\right)$ is the bilateral PSD at the output of the input mixer, i.e.,

(13) $$S_{AMP}\left(f\right)=\frac{1}{2}{\left(\frac{C_{IN}+C_{p}}{C_{IN}}\right)}^{2}S_{n,in,OTA}\left(f\right)$$

The factor 1/2 is due to the passage from the unilateral spectrum (1) to the bilateral spectrum in chopping theory (6).

Without the chopper, (1) results in

(14) $$V_{n,in,LNA}^{2}=\int_{F_{L}}^{F_{H}}S_{AMP}\left(f\right)\;df=2N_{th}\left(B_{N}+f_{nc}ln\frac{F_{H}}{F_{L}}\right)$$

where $B_{N}=F_{H}-F_{L}$ is the bandwidth of interest and $F_{H}=f_{ch}/2$.

On the other hand, the input-referred noise when the CS approach is used can be obtained starting from (12), integrating over the bandwidth of interest, and dividing by the squared gain $A_{0}^{2}$ ($A_{0}$ includes the input capacitive divider),

(15) $$V_{n,in,chop}^{2}=\frac{2}{A_{0\;}^{2}}\int_{F_{L}}^{F_{H}}S_{N_{0}}\;\left(f\right)df=\frac{16}{\pi^{2}}N_{th\;}\zeta_{F}\;B_{N}$$

with $\zeta_{F}\leq \pi^{2}/8$. A comparison of (14) and (15) shows that in the conventional approach flicker is present and is the dominant source of the input referred noise, thus noise optimization mainly involves its minimization by increasing the size of the input devices, whereas in the CS approach flicker is ideally removed (at least if $f_{nc}<f_{ch}/2$); therefore, integrated noise is lower for the same thermal noise level, and optimization is focused on thermal noise.

#### 3.3.3. Design Flow for the Proposed CS-LNA

We have demonstrated that the use of the CS approach makes flicker noise of the OTA negligible. The design procedure is thus no more focused on minimizing it, as in [24], and a different design flow can be devised for LNAs that exploit chopper stabilization. Design goals must include gain and bandwidth, minimization of noise power and silicon area, as well as the minimization of the intrinsic offset voltage of the OTA. In fact, it has to be noted that, while the proposed CS approach rejects the EDO and the flicker noise, the intrinsic offset of the OTA, mainly due to mismatch in the input stage, results in an incorrect biasing of the OTA, with a reduction in the output voltage swing of the amplifier. Offset standard deviation is inversely proportional to the square root of device area; hence, offset minimization involves an increase of $W_{1,2}L_{1,2}$. However, the area required to ensure an acceptable value of the output offset voltage is typically much lower than that required to make flicker noise negligible with respect to thermal noise.

These considerations allow for defining a design flow for the CS-LNA, that does not take into account flicker noise contribution (we assume $f_{ch}>2f_{nc}$). The starting point is the minimization of the thermal noise of the OTA, given by (3), that depends on the factor *γ* and on the transconductance *gm*_1,2_ of the input pair devices. The transistors of the input pair have to operate in weak inversion, to maximize their transconductance for a given current and satisfy Condition (5) with suitable aspect ratios; their transconductance is therefore directly proportional to the bias current. The factor *γ* can be shown to be dependent on the $gm/I_{D}$ ratio [41], and tends to saturate for high ratios (hence transistor biased well below the threshold). It is thus possible to select a suitable value of $gm/I_{D}$, as a trade-off between low *γ* and non-excessive aspect ratio *W*/*L*, and hence thermal noise specifications determine both the bias current *I_TAIL_* and the aspect ratio $S_{1,2}=W_{1,2}/L_{1,2}$ of the input transistors of the OTA. The values of gate width and length can then be determined by taking into account the minimization of the offset, that sets the minimum value for the gate area $W_{1,2}L_{1,2}$.

The remaining specifications determine the sizing of the other transistors. The voltage gain $A_{D}$ of OTA in Figure 4a can be easily found to be

(16) $$A_{D}=g_{m1,2}R_{OUT}$$

where $R_{OUT}$ is the equivalent resistance at the output of the OTA, which can be expressed as

(17) $$R_{OUT}≅\left(r_{o1,2}g_{m3,4}r_{o3,4}\right)||\left(r_{o7,8}g_{m5,6}r_{o5,6}\right)$$

where usual notation for small signal parameters of MOS devices is adopted. Since $R_{OUT}$ depends on the bias current $I_{D3,4}$ in the output branches, a specification on $A_{D}$ can be easily translated into a requirement for $I_{D3,4}$, and therefore for $I_{STEAL}$. Then, once chosen, the desired $gm/I_{D}$, the aspect ratio S=W/L of the transistors in the output branches can be found.

The gain of the proposed CS-LNA can be controlled by modifying the value of the voltage $V_{TUNE}$ (see Figure 4a), thus changing the value of $R_{OUT}$ through a variation in $r_{o3,4}$ in (17). This approach allows a gain tuning range in the order of 10 to 15 dB.

The bandwidth of the OTA in Figure 4a can be expressed as

(18) $${BW}_{OTA}=\frac{1}{2\pi \;{R_{OUT}\;C}_{L}}$$

where $C_{L}$ is the load capacitance of the OTA. The requirement on the bandwidth therefore sets the maximum load capacitance as follows:

(19) $$C_{L,max}=\frac{1}{{2\pi \;R_{OUT}\;BW}_{OTA}}$$

## 4. Design of the CS-LNA Prototype in 130 nm CMOS Technology

The proposed CS-LNA has been designed and fabricated in the STMicroelectronics 130 nm CMOS technology as a first prototype of an in-pixel amplifier for a multi-channel neural recording system. The open-loop OTA, reported in Figure 4, has been designed to process signals from DC to about 15 kHz, while rejecting the EDO trough the DSL-free approach described in Section 2. In order to reduce the IRN by keeping the area compatible with the in-pixel application, the optimization approach exploiting the chopper stabilization technique discussed in Section 3 has been adopted. In particular, from the specification on thermal noise, ${gm}_{1,2}$ and ${gm}_{1,2}/I_{D1,2}$ have been set to about 28 μS and 28 $V^{-1}\;$ respectively, with $I_{D1,2}=1$ μA. For a voltage gain in the range of 40 dB, from (16) $R_{OUT}$ is found to be about 3.5 MΩ. Then, starting from (17), the bias current $I_{D3,4}$ in the output branches has been set to 10 nA. For ${BW}_{OTA}$ = 15 kHz, the maximum load capacitance has been found to be $C_{L,max}≅3$ PF, which is much higher than the input capacitance of the next stage. From preliminary Monte Carlo simulations, the value of $W_{1,2}L_{1,2}$ that guarantees an input offset voltage standard deviation lower than 1 mV has been found to be $W_{1,2}L_{1,2}$ = 200 μm^2^, resulting in a parasitic capacitance $C_{p}$ in the order of about 0.2 pF. C_IN_ has then been set to 10 pF, which is high enough to make the term ${\left(\frac{C_{IN}+C_{p}}{C_{IN}}\right)}^{2}$ in Equation (1) about equal to unity.

With the above design choices, the noise corner frequency $f_{nc}$ of the OTA results in being about 1 kHz, and the chopping frequency has been allowed to vary from 8 to 15 kHz in order to guarantee $f_{nc}<f_{ch}/2$ in all cases, and to push the flicker noise out of the required signal bandwidth.

The values of main parameters for the transistors in Figure 4a are summarized in Table 1, for $I_{BIAS}$ = 2 μA, and $V_{TUNE}=0.1\;V$.

The common-mode feedback (CMFB) loop in Figure 4b has been implemented by using a simple PMOS differential pair with an active load and biased with a tail current of 10 nA.

The supply voltage V_DD_ of the LNA has been set to 0.8 V for an overall power consumption of about 1.6 μW, resulting in a good trade-off for ultra-low power high-density recording sites.

The CMFB loop allows to accurately set the common-mode output voltage of the OTA at 0.4 V, thus maximizing the output voltage swing. Each transistor in the chopper mixers in Figure 5 has been sized to minimize the noise contributions with *W/L* equal to 5/0.13 μm. The same sizing has been adopted for the TG switch driven by the *F_RESET_* signal (see Figure 2 and Figure 3).

## 5. Experimental Results

**Figure 7:**
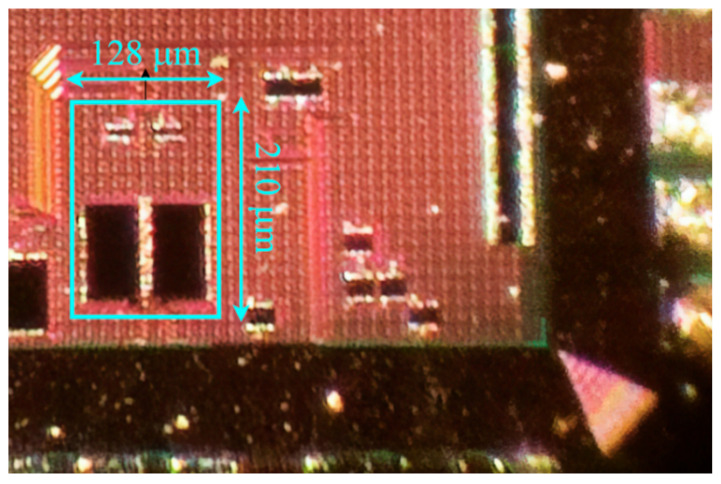
Die microphotograph showing the detail of the in-pixel LNA prototype.

The die microphotograph of the test chip showing the detail of the in-pixel LNA is reported in Figure 7. The CS-LNA occupies an area of 0.027 mm^2^. The fabricated test chip has been packaged, and a dedicated test-board has been developed for the characterization.

### 5.1. Simulations

Periodic steady state (PSS) and periodic AC (PAC) simulations in the Cadence Virtuoso environment have been exploited to analyze the real capability of the adopted chopper-based, DC servo-loop-free approach, to reject the EDO. The values of the sensing electrode parameters assumed for the simulations are *R_SS_* = 8 kΩ, *R_PS_* = 400 MΩ, and *C_PS_* = 50 nF, whereas the values adopted to model the reference electrode are *R_PF_ =* 50 kΩ, *R_SF_* = 50 MΩ, and *C_PF_* = 400 nF. These values, taken from [30,31], refer to a microelectrode coated with Pt-black of the dimensions equal to 900 μm^2^.

The chopper signal frequency *F_CHOP_* and the reset signal frequency *F_RESET_* have both been set at 10 kHz, which is high enough to allow the detection of the LFP and AP signals.

**Figure 8:**
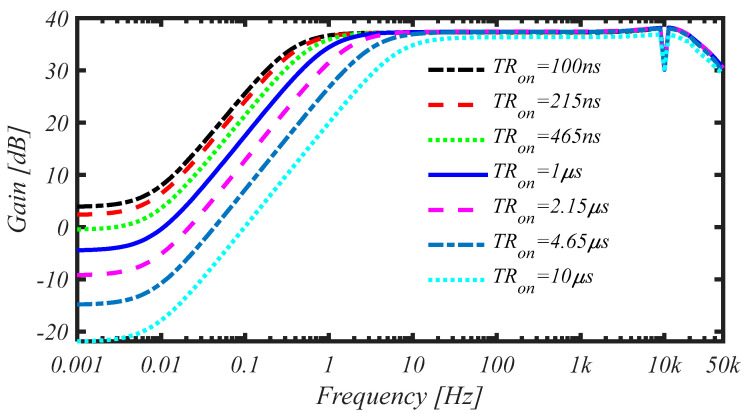
Frequency response of the CS-LNA for different values of ${TR}_{on}$ from PAC simulation.

The duty-cycle of the reset signal has been varied from 0.1% to 10%, resulting in a reset time ${TR}_{on}$ ranging from 100 ns to 10 μs. Results of the PAC simulations showing the frequency response of the CS-LNA for the different values of ${TR}_{on}$ are reported in Figure 8.

By looking at Figure 8, it can be noted the modulation of the high-pass pole providing a maximum DC rejection ranging from a minimum of 30 dB up to a maximum of about 58 dB. The results obtained from a transient simulation for ${TR}_{on}$ = 1 μs have confirmed that the adopted approach allows to tolerate an input EDO of ±50 mV, without appreciably affecting the CS-LNA performance. Similar results ore obtained for *F_CHOP_* ranging from 8 kHz to 15 kHz.

To assess the robustness of the proposed CS-LNA, we have carried out also corner and mismatch Monte Carlo simulations. Results of corner simulations are summarized in Table 2, where Z_IN_ (MΩ) denotes the magnitude of input impedance at the frequency of 1 kHz, *CMRR* is the common-mode rejection ratio at 1 Hz, *PSRR* is the power supply rejection ratio at 1 Hz, and *THD* is the total harmonic distortion at 1 mVp. Temperature variations have not been considered because, in implanted devices, very small temperature variations are allowed [10].

The corners TT, FF, SS, FS, and SF refer to MOS devices, as usual, whereas for MIM capacitors the maximum capacitance has been associated with the SS and SF corners, and the minimum capacitance with the FF and FS corners.

**Figure 9:**
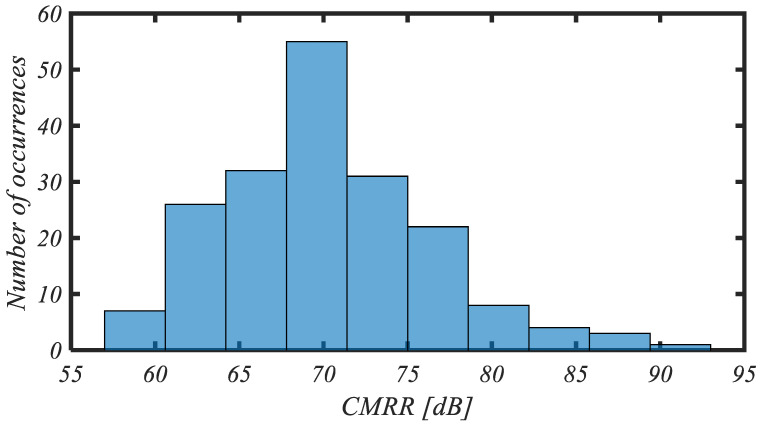
Histogram of CMRR for 200 mismatch Monte Carlo iterations.

The histogram of the *CMRR* at 1 Hz for 200 mismatch Monte Carlo iterations is reported in Figure 9. The mean value and standard deviation of the *CMRR* are 70.2 dB and 6.2 dB, respectively.

**Figure 10:**
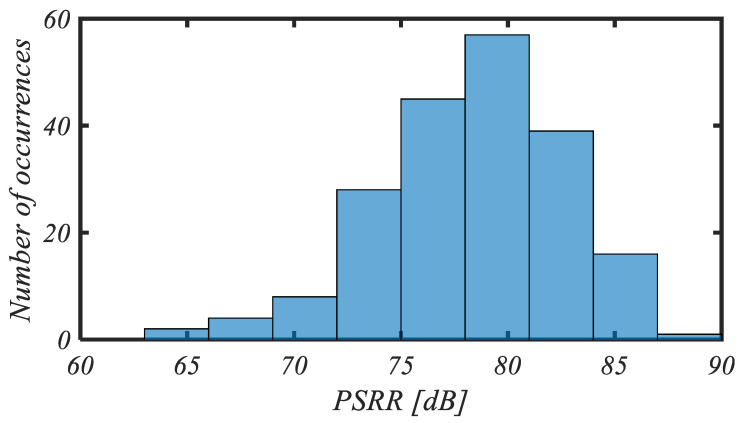
Histogram of PSRR for 200 mismatch Monte Carlo iterations.

The histogram of the *PSRR* at 1 Hz for 200 mismatch Monte Carlo iterations is reported in Figure 10. The mean value and standard deviation of the *PSRR* are 78.3 dB and 4.8 dB, respectively.

**Figure 11:**
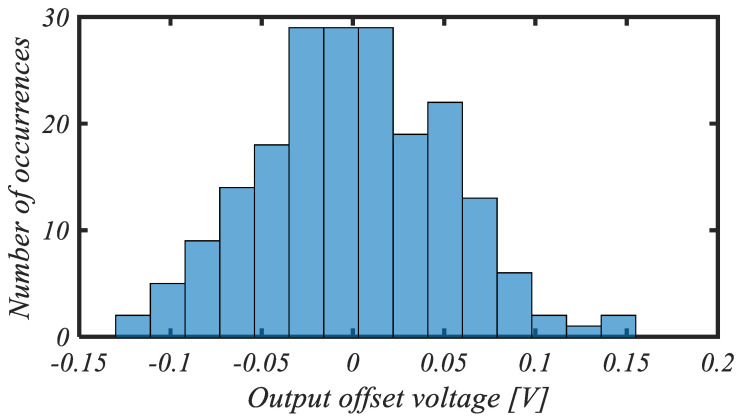
Histogram of output offset voltage for 200 mismatch Monte Carlo iterations.

The histogram of the intrinsic DC differential output voltage offset for 200 mismatch Monte Carlo iterations is reported in Figure 11. The mean value of the output offset is very close to 0 V, as expected, whereas the standard deviation of the output offset is 51.3 mV, which, for a voltage gain in the range of 38 dB, results in an input-referred offset standard deviation of about 0.7 mV.

### 5.2. Measurements

**Figure 12:**
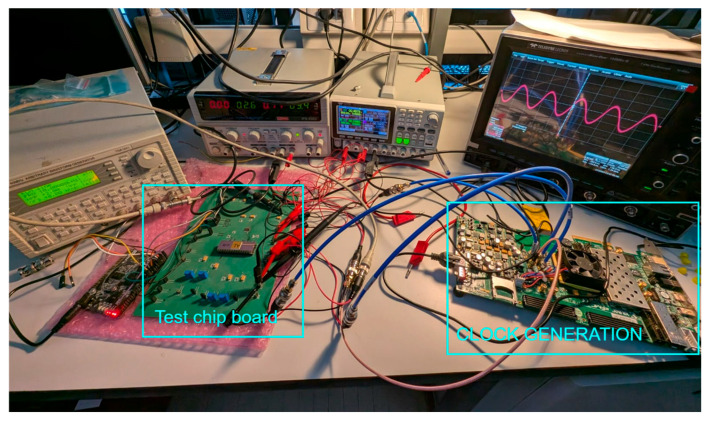
Photo of the measurement setup showing the test chip board and an FPGA board for clock generation, together with an arbitrary waveform generator and a digital oscilloscope.

The measurement setup including the test-chip board, and FPGA board for clock generation is reported in Figure 12.

**Figure 13:**
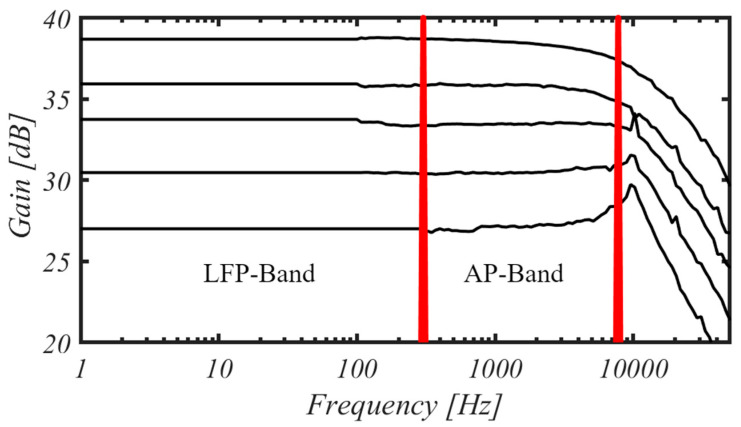
Measured LNA gain with programmable range from about 27 to 39 dB. (Chopping frequency set at 9 kHz. Minimum measured frequency is 1 Hz due to measurement setup limitations).

The measured mid-band gain of the proposed in-pixel amplifier is reported in Figure 13 for a chopping frequency of 9 kHz, showing a gain programmability from 27 to 38.67 dB, and a useful bandwidth approaching the chopping frequency. The minimum measured frequency is 1 Hz due to measurement setup limitations.

**Figure 14:**
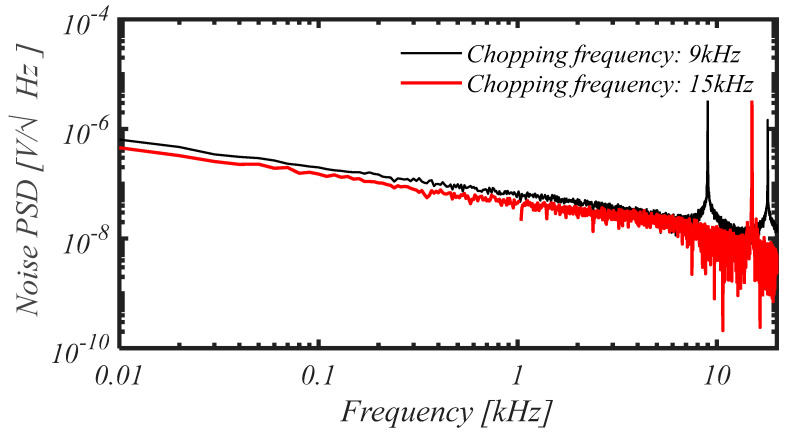
Measured input noise PSD for chopping frequency set at 9 kHz (black trace) and at 15 kHz (red trace).

The measured input noise PSD for chopping frequency set at 9 kHz (black trace) and at 15 kHz (red trace) is reported in Figure 14, showing a spot noise at 1 kHz equal to $66\;nV/\sqrt{Hz}$ and $44\;nV/\sqrt{Hz}$, respectively.

For a chopping frequency of 15 kHz, the noise integrated from 1 Hz to 7.5 kHz resulted 4.19 μ$V_{RMS}$, whereas integrated in the LFP band from 1 Hz to 300 Hz and in the AP band from 300 Hz to 7.5 kHz resulted to be 2.93 μ$V_{RMS}\;$ and 2.58 μ$V_{RMS}$, respectively.

The common-mode gain measured at 1 Hz for ${TR}_{on}$ = 1 μs has resulted to be about −28 dB, resulting in a CMRR of about 67 dB, in reasonable agreement with the average value from Monte Carlo simulations. Similar results have been obtained on a second available packaged chip.

**Figure 15:**
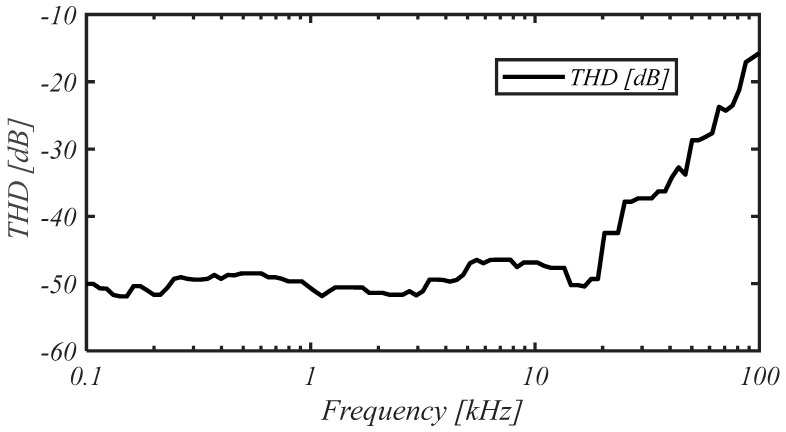
Measured THD vs. frequency for an input amplitude of 1 mV.

**Figure 16:**
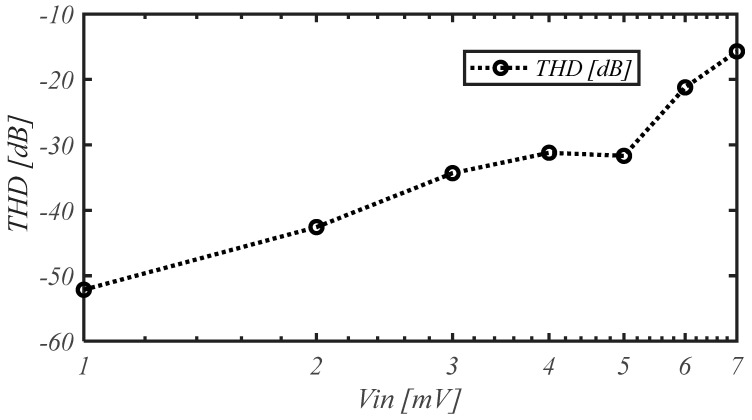
Measured THD vs. input amplitude at the frequency of 1 kHz.

The total harmonic distortion (THD) vs. frequency for an input amplitude of 1 mV is reported in Figure 15, whereas the THD vs. the amplitude of the input signal at the frequency of 1 kHz is shown in Figure 16, highlighting a THD better than −40 dB (1%) for input amplitudes of about 2.5 mV.

A comparison table showing the main performance figures for recent in-pixel LNAs for neural recording applications is reported in Table 3. For the comparison, the following metrics are used:

(20) $$NEF=IRN\cdot \sqrt{\frac{2I_{TOT}}{\pi \cdot V_{T}\cdot 4kT\cdot BW}}$$

where *IRN* is the total input-referred noise, Itot is the total supply current, *V_T_* is the thermal voltage at absolute temperature *T*, and *BW* is the −3 dB bandwidth of the amplifier.

(21) $$PEF={\left(NEF\right)}^{2}\cdot V_{DD}$$

where $V_{DD}$ is the supply voltage of the amplifier.

(22) FoM=PEF·AREA

where *AREA* is the silicon area occupied by the amplifier expressed in mm^2^.

Results reported in Table 3 show that the proposed LNA exhibits the best performance in terms of IRN and NEF in the AP bandwidth, the lowest supply voltage, and overall performance in line with the state of the art.

The comparison in terms of the FoM reported in Equation (22) confirm that the proposed CS-LNA provides the best tradeoff in terms of noise, area and power consumption.

## 6. Conclusions

This paper proposes and experimentally validates an ultra-low-power variable gain amplifier for neural recording applications in the LFP and AP bands, which exploits chopper stabilization (CS) to minimize the impact of flicker noise, and an open-loop architecture to minimize area and power consumption.

Furthermore, the paper provides a design flow to optimize the amplifier, once noise, mismatch, and power consumption constraints have been set. The design flow is based on the hypothesis that CS amplifiers require no minimization of flicker noise, so that only white noise shall be optimized.

The proposed amplifier has the lowest supply voltage, one of the lowest power consumptions (especially compared with the bandwidth), and the best noise performance in terms of NEF. It exhibits also the lowest integrated noise in the AP bandwidth. CMRR data show the robustness of the design. Experimental results and Monte Carlo simulations show remarkable performance and robustness.

## Figures and Tables

**Table 1:** Device sizing and design parameters.

Device	W/L [μm]	$g_{m}$ [*S*]	$g_{m}/I_{D}\left[V^{-1}\right]$
M0	100/2	47 μ	23.5
M1–M2	200/1	28 μ	28
M_3_–M_4_	1/10	176 n	17.6
M_5_–M_6_	0.5/10	233 n	23.3
M_7_–M_8_	5/50	255 n	25.5
M_9_–M_10_	5.1/20	10.7 μ	10.7
M_11_	100/2	47 μ	23.5
M_12_–M_13_	5.1/20	10.7 μ	10.7

**Table 2:** LNA performance in the different process corners for ($V_{TUNE}=0.1V)$.

Corner	*Gain* (dB)	*Z*_*IN*_ (MΩ) at 1 kHz	*CMRR* (dB)	*PSRR* (dB)	*THD* (%) at 1 mVp
TT	35.5	10.75	70.0	85.9	0.055
FF	34.6	11.67	75.5	87.6	0.047
SS	35.8	10.13	65.9	63.9	0.036
FS	34.5	11.53	60.2	80	0.021
SF	35.7	10.03	74.7	89.7	0.060

**Table 3:** Performance comparison.

References	This Work	[19]	[20]	[27]	[15]	[24]	[42]	[28]
Technology (nm)	130	65	180	180	180	130	180	40
Applications	LFP + AP	LFP	LFP + AP	LFP + AP	LFP + AP	LFP + AP	AP	LFP + AP
Supply Voltage (V)	0.8	1.2	1	1.8	1.2	1.5	1.2	1.2
Gain (dB)	27–39	53–61	37	20–40	43–63	37	40	26
BW (kHz)	7.5	0.5	7.1	10	10	7	5	4.8
CMRR (dB)	67	-	75	77	60	-	70	-
Power (μW)	1.6	2.78	1.2	13.7	8.57	1.5	1.28	2
IRN (μ$V_{RMS}$)								
Full BW (1 Hz–7.5 kHz)	4.19	-	-	-	6.32 ^2^	-	-	-
LFP (1 Hz–300 Hz)	2.93	0.88	-	1.32	-	-	-	2
AP (300 Hz–7.5 kHz)	2.58	-	5.3	3.36	-	5.5	3 ^1^	7
NEF (AP)	1.62	-	2.65	4.77	-	2.58	1.68 ^1^	4.9
PEF (AP)	2.099		7.022	40.95		9.9846	3.39	28.81
$\mathrm{Area}\;(mm^{2}$)	0.027	0.14	0.012	0.036	0.016	-	0.030	0.071
FoM=PEF·Area$\;(mm^{2}$)	0.056	-	0.084	1.474	-	-	0.101	2.045

^1^ Integrated from 600 Hz to 5 kHz. ^2^ Integrated from 0.5 Hz to 10 kHz.

## Data Availability

Data are contained within the article.
